# Zoonotic attack: An underestimated threat of SARS-CoV-2?

**DOI:** 10.1016/j.xinn.2022.100242

**Published:** 2022-04-15

**Authors:** Lin Zhu, Honglin Chen, Zongwei Cai

**Affiliations:** 1 State Key Laboratory of Environmental and Biological Analysis, Hong Kong Baptist University, Hong Kong SAR, China; 2 Department of Microbiology and State Key Laboratory for Emerging Infectious Diseases, Li Ka Shing Faculty of Medicine, The University of Hong Kong, Hong Kong SAR, China

As the first coronavirus pandemic is walking into its third year, it is likely that the pandemic will transform to an endemic disease soon. With global efforts and measures on mass immunization, non-pharmaceutical interventions, and therapeutics advancements, the scales of death, illness, or social isolation have been relieved. However, endemic does not mean harmless; rather, it could be widespread and deadly.^1^

Ever since its emergence in 2019, SARS-CoV-2 has been mutating and evolving, leading to recent variant Omicron with significant transmissibility and immune-escaping capacities. Current vaccines could alleviate severe diseases but might not prevent infection and block the transmission. Growing genetic diversity of SARS-CoV-2 and large population of infection have increased the frequency of coinfection by distinct viral lineages, consequently leading to meaningful recombination events, generating novel recombinants with potential impact. In fact, multiple recombinant strains have been reported worldwide recently. XE, a recombinant of Omicron sublineages BA.1 and BA.2, was reported to have 9.8% relative growth advantage over the current major variant BA.2.^2^ It is likely that new variants will continue to generate, leading to challenges in public health measurements.

A recent study reported multiple zoonotic transmission events from pet hamster to human, revealing a worrying loophole in current pandemic control and surveillance systems.^2^ A delta variant of concern (VOC) circulated among pet hamsters and transmitted to human, causing a local outbreak in Hong Kong. Phylogenetic and epidemiological analyses indicated that the virus had infected and persisted among hamsters prior to importation, which later became the source of local transmission of Delta.^3^

**Figure 1:**
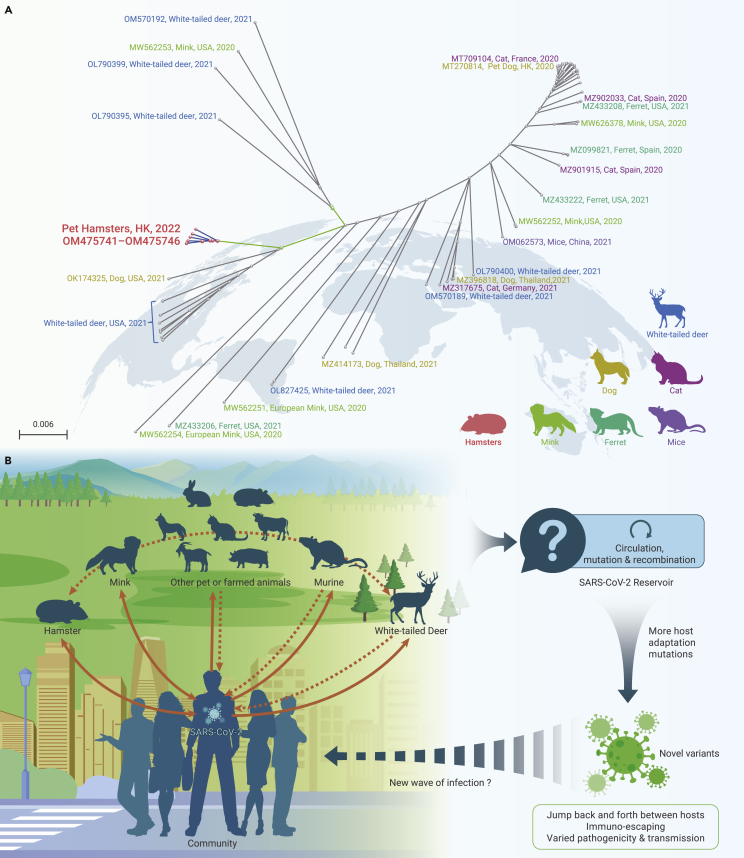
Phylogenetic tree of SARS-CoV-2 isolated from animal hosts and the proposed transmission network (A) Phylogenetic tree of SARS-CoV-2 strains isolated from animal hosts. A total 69 whole genome sequences were obtained from NCBI and plotted in the layout of radial tree with midpoint root. Six sequences of SARS-CoV-2 isolated from pet hamsters were marked in red. (B) A potential transmission network of SARS-CoV-2. Blue arrow represents the direction of transmission. Solid lines represent documented transmission route. Dash lines represent proposed transmission that have not been reported so far.

It is believed that SARS-CoV-2 might have originated from bat and acquired its ability to infect humans via mutations on spike protein.^4^ Prior to the reported hamster-to-human transmission, mammals in the wild or on farms or as pets have been reported to be infected by different SARS-CoV-2 lineages via human contact. Continuous evolution of human SARS-CoV-2 was observed in mink during the mink farm outbreak and in wild white-tailed deer, which were caused by selection pressure in the new hosts. A phylogenetic tree was plotted using 69 whole viral genome sequences isolated from different animal hosts deposited in NCBI SARS-CoV-2 Data Hub (Figure 1A). A great variety of PANGOLIN lineages was found, suggesting spillovers from human to animal were relatively easy and frequent.

However, these spillover events, except the one in mink farms, were only one-way transmission and could not occur from animals to human. The reason might either be a mutation that disabled the infectivity of the virus to humans under selection pressure of animal hosts or an unfavorable environment in the new host to generate enough infectious virions. In the case of the mink farm, the transmission probably occurred due to high-dose environmental exposure of virus with high density of animals.^4^

Therefore, the reported transmission of pet hamster to human was particularly worrisome: it showed how easy it would be for the virus to be transferred across international borders via pet commerce, while international travel for humans is still under tight control. Given that pet animals including dog, cat, hamster, etc., have been reported to be infected by SARS-CoV-2, the lack of surveillance in the pet industry would be a potential loophole for prevention and control of COVID-19. This incident also confirmed the abilities of the virus to circulate, persist, and mutate among hamsters, yet remain in its competency to infect humans. It not only meant the virus could continue to evolve and spillover back to humans, but also indicated an emerging bigger issue. The possibility of coronavirus to acquire a key host adaptation mutation to enable it to jump back and forth between humans and animals might increase (Figure 1A).

The host tropism of SARS-CoV-2 has continuously expanded by acquiring different host adaptation mutations. N501Y mutation existing in multiple VOCs enables the virus to infect and reproduce in wild murine.^5^ Considering the wide spread of VOCs carrying N501Y that has been detected in both humans and environments, an undiscovered circulation of SARS-CoV-2 among murine and even other zoonotic species may exist, constituting a serious challenge to public health.

What is more, reported incidents of SARS-CoV-2 infection in animals and related zoonotic transmissions may just be a tip of the iceberg due to limited surveillance. It is known that the virus may circulate among wild white-tailed deer. New variants carrying host adaptation mutations may appear and transmit to other wild animals. The expansion of host tropism of coronavirus may bring novel variants with unknown threats to humans, including significant immune-escaping property, increased transmissibility, and the varied pathogenesis.

Another threat in formation but rarely being brought up is the establishment of SARS-CoV-2 reservoir in wild animals. Influenza A virus, for example, primarily relies on wild birds as its natural reservoir and continuously serves as gene pools to influenza viruses in domestic poultry and swine. Cross-species transmission from animals to human caused by host adaptation mutations was believed to lead to the 1918 flu pandemic and a number of pandemics later on, including the 2009 swine flu pandemic. A similar scenario might also happen for SARS-CoV-2 if the coronavirus could persist and circulate in wild animals globally, which would increase the difficulty for monitoring. Given the frequent recombination of coronaviruses, novel recombinant viruses might appear by recombination between SARS-CoV-2 and other coronaviruses that already circulate among wild animals (Figure 1B). Potential threats of recombination between distinct lineages were already suggested by the appearance of recombinant XD, XF, and XE.^2^ However, very few efforts are made currently to monitor possible recombination events in the animal population.

Therefore, even though the COVID-19 pandemic may end in the near future, there is still much to do for public health management. Systemic surveillance and monitoring for SARS-CoV-2 existing in wild, farmed, and pet animals are of crucial importance. Preparation for investigation and production of vaccine based on newly detected variants may be considered. Additional measures for regulating and monitoring the pet trade seem to be necessary, while policy makers should take extra care when making decisions and adopting guidelines regarding the management of susceptible pet animals due to the unique and emotional bond between pets and their owners.
